# MVP and vaults: a role in the radiation response

**DOI:** 10.1186/1748-717X-6-148

**Published:** 2011-10-31

**Authors:** Pedro C Lara, Martin Pruschy, Martina Zimmermann, Luis Alberto Henríquez-Hernández

**Affiliations:** 1 Radiation Oncology Department, Hospital Universitario de Gran Canaria Dr Negrín. C/Barranco de La Ballena s/n, 35010, Las Palmas de Gran Canaria, Spain; 2 Clinical Sciences Department, Universidad de Las Palmas de Gran Canaria. C/Dr. Pasteur s/n, 35016, Las Palmas de Gran Canaria, Spain; 3 Instituto Canario de Investigación del Cáncer, Canary Islands, Spain; 4 Radiation Oncology Department, University Hospital Zürich. Raemistrasse 100CH-8091, Zürich, Switzerland

**Keywords:** major vault protein, radiotherapy, prognosis, radiation response

## Abstract

Vaults are evolutionary highly conserved ribonucleoproteins particles with a hollow barrel-like structure. The main component of vaults represents the 110 kDa major vault protein (MVP), whereas two minor vaults proteins comprise the 193 kDa vault poly(ADP-ribose) polymerase (vPARP) and the 240 kDa telomerase-associated protein-1 (TEP-1). Additionally, at least one small and untranslated RNA is found as a constitutive component. MVP seems to play an important role in the development of multidrug resistance. This particle has also been implicated in the regulation of several cellular processes including transport mechanisms, signal transmission and immune responses. Vaults are considered a prognostic marker for different cancer types. The level of MVP expression predicts the clinical outcome after chemotherapy in different tumour types. Recently, new roles have been assigned to MVP and vaults including the association with the insulin-like growth factor-1, hypoxia-inducible factor-1alpha, and the two major DNA double-strand break repair machineries: non-homologous endjoining and homologous recombination. Furthermore, MVP has been proposed as a useful prognostic factor associated with radiotherapy resistance. Here, we review these novel actions of vaults and discuss a putative role of MVP and vaults in the response to radiotherapy.

## Review

### Major vault protein: an overview of structure and composition

**Figure 1:**
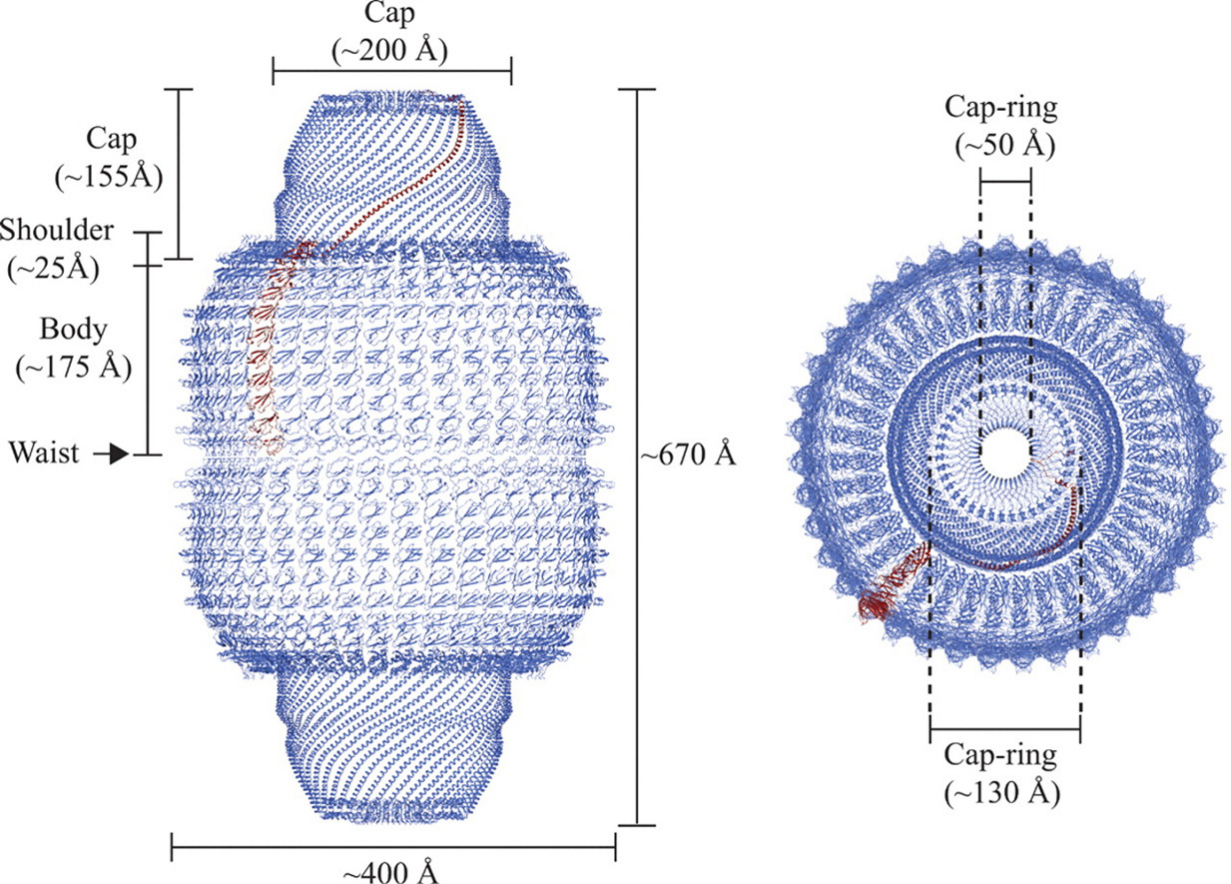
**Overall structure of the vault shell**. One molecule of MVP is colored in tan, and the others are colored in purple. (Left) Side view of the ribbon representation. The whole vault shell comprises a 78-oligomer polymer of MVP molecules. The size of the whole particle is ~670 Å from the top to the bottom and ~400 Å in maximum diameter. The particle has two protruding caps, two shoulders, and a body with an invaginated waist. Two half-vaults are associated at the waist with N-terminal domains of MVP. (Right) Top view of the ribbon representation. The maximum diameter of the cap is ~200 Å. The outer and the inner diameters of the cap-ring are shown. Figure reproduced from Tanaka *et al*. (2009) with permission from The American Association for the Advancement of Science (Sciences Magazine).

Vaults are ribonucleoprotein particles with a hollow barrel-like structure [1] and a mass of 13 MD. In mammals, it is composed of three proteins: MVP (104 kD), the vault poly(adenosine diphosphate-ribose) polymerase also known as VPARP (193 kD), and telomerase-associated protein-1 TEP1 (240 kD), and small untranslated RNA (vRNA) of 141 bases. MVP constitutes more than 70% of the total mass of the complex [2-4], while vARN represents less than 5% [5]. The molecular architecture of the rat liver vault complex was recently elucidated at high resolution [6]. A vault consists of 2 dimers of half-vaults, which align at their waists to form together a barrel-like structure with the overall dimensions of 72 × 41 × 41 nm. Each half-vault comprises 39 identical major vault proteins (MVP), the major self-assembling structural component (Figure 1). Interestingly, vaults can open, the two halves can dissociate at their waists at acidic pH, and half vaults can be exchanged to form new vaults. Based on these features and on its large interior volume, which may encapsulate hundreds of proteins, recent interest in recombinant vaults derives from nanoparticle research trying to exploit vaults as drug delivery system [7,8].

The sequences of the 2 other proteins, which are not part of this shell-like structure and probably reside at the top center of the caps or within the vaults, are identified and are present also in the human genome. VPARP presumably ribosylates substrates and TEP1 is important for stabilization of vRNA. Molecular composition of the vault has been roughly estimated as 78-96 MVPs, eight VPARPs, two TEP1s, and at least six copies of vRNA [9]. Both the high degree of evolutionary conservation and the complex structure of vault particles, as well as its broad distribution in tissues, suggest an important function in cellular processes [10]. Although vaults have been proposed to play a role in drug resistance, nucleocytoplasmic transport, and regulation of signaling, a definitive function for MVP or vaults has yet to be assigned as MVP knockout mice (MVP^-/-^) do not have phenotypes consistent with these in vitro observations [11]. This suggests that even though the major component of the vault particle is absent in MVP^-/-^-mice, and vault particles are no longer detected, the remaining components TEP1, VPARP, and vRNA might still interact and possibly fulfill a functional role.

The human gene encoding MVP has been located in chromosome 16 (16p11.2) [12], approximately 27 cM proximal to the gene location of the multidrug resistance protein-1 (MRP1, also designated as ABCC1) [12]. However, although both the ABCC1 and MVP maps to the short arm of chromosome 16, they are rarely coamplified and are normally not located within the same amplicon and can be switched on separately [12,13].

Analysis of the human MVP gene revealed a TATA-less promoter, which also lacks other core-promoter elements but harbors several putative transcription factor binding sites, including an inverted CCAAT box, a p53-binding site, and a GC box element [14]. In silico analysis identified a putative STAT-binding site that strongly resembles an interferon-γ-activated site element (GAS), which binds preferentially to STAT1 homodimers [15]. Disruption of the STAT-binding site reduces basal MVP promoter activity, suggesting a role of JAK/STAT signals in the activation of MVP expression [16]. With up to 10^5 ^particles per cell, vaults are abundantly present in many different cell types, although its expression varies among tissues. Vaults are most numerous in macrophages [17,18] and epithelial cells with secretory and excretory functions as well as cells chronically exposed to xenobiotics such as bronchial cells and cells lining the intestine [19]. Most of the vaults are located in the cytoplasm, although a small fraction of these particles apparently is also localized at or near the nuclear membrane and the nuclear pore complex [20,21]. The widespread of vaults in many diverse organisms and cell types suggests that their function is essential to eukaryotic cells.

### Interaction Partners and Putative Functions of Vaults

The detailed insight on the structure of MVP and vaults contrasts with the poor understanding of their endogenous function. Several observations though indicate that MVP is directly involved in major cytoplasmic signal transduction cascades. In EGF-stimulated cells, tyrosine-phosphorylated MVP complexes with the SH2-domain-containing tyrosine phosphatase SHP-2 and interacts with ras-activated downstream kinase Erk, presumably to fine-tune the activity of the Erk-downstream target Elk-1 [22]. But tyrosine-phosphorylated MVP also binds to the SH2-domain of Src-kinase and downregulates EGF-dependent ERK activation in Src overexpressing cells [23]. Likewise MVP also binds to the tumour suppressor phosphatase and tensin homolog deleted on chromosome 10 (PTEN) and a significant quantity of endogenous PTEN associates with vault particles in HeLa cells, thus suggesting a regulatory role for MVP in the phosphoinositide 3-kinase/Akt (PI 3-kinase/Akt) signaling pathway [24]. However, MVP-PTEN-interaction might only be relevant for nuclear import of PTEN thereby regulating the nuclear function of PTEN ([25], *see below*). Eventually, the identification of the specific growth factor-regulated MVP phosphorylation sites and the complexed interaction partner on MVP in response to growth factor stimulation are required to reveal a scaffold or direct regulatory function of MVP/vaults on these signal transduction cascades.

Likewise, a co-immunoprecipitation approach revealed that the UV-induced phosphorylation status of MVP affects the capacity of the constitutive photomorphogenic 1 ubiquitin ligase (COP1) to interact with the transcription factor c-jun. In unstressed cells, cytoplasmic MVP/vault binds to and modifies COP1, presumably via the other vault components. Following shuttling into the nucleus, COP1 complexes with c-jun with high affinity and suppresses its transcriptional activity by targeting c-jun for degradation. After UV irradiation, phosphorylated MVP does not interact with COP1 anymore, eventually leading to the alleviation of COP1-c-jun- complex formation. Free c-jun subsequently drives AP-1-controlled gene expression [26]. Negative regulation of downstream signaling has also been identified on the level of the hypoxia-inducible factor-1α (HIF-1). HIF-1α and MVP-expression are increased under hypoxia. However, the stability of HIF-1α protein is enhanced to higher extents in MVP-knockdown cells. Direct complex formation of vaults with HIF-1α, PHDs and pVHL was demonstrated and suggests that MVP/vault might act as scaffold protein for ubiquitination and degradation of HIF1α [27] and thereby affects hypoxia-regulated expression of HIF-1α-dependent gene expression.

The generation of MVP^-/-^-mice did not reveal any further indications on an essential role of MVP and vaults during embryogenesis and in the cellular processes in tissues of adult mice under normal environmental conditions. Furthermore, both embryonic stem cells and bone marrow cells derived from MVP^-/-^-mice display a similar sensitivity towards classic cytostatic agents as their wildtype counterpart cells, and normal tissue toxicity towards doxorubicin in vivo is unchanged in the MVP-deficient mice. Interestingly though and unexpectedly identified, MVP and vaults are part of the effective innate immune response and contribute to the uptake and clearance of lung pathogens such as Pseudomonas aeruginosa via lipid rafts in lung epithelial cells, and eventually resistance to lethality [28]. Furthermore, the activation of MVP-expression by interferon γ through the JAK/STAT pathway and enhanced expression of MVP in macrophages and in dendritic cells suggest a clear relationship with the immune system [16,29]. However, despite the high abundance of MVP/vaults in MVP-wildtype dendritic cells, no differences in dendritic cell migration and antigen presentation and T-cell responses could be observed in MVP^-/-^-mice. In human beings, a strong up-regulation of vault expression during dentritic cell culturing was observed, proposing that vaults act as components in the cascade of events regulating dentritic cell effector function [30]. Besides the activation of MVP by interferon, several other observations indicate that MVP might be involved in immunological responses [31]. It has been reported a dramatic induction of vRNA expression in response to infection with Epstein-Barr virus [32,33], and vRNA induction has been has been suggested as playing important roles in innate immunity [34]. This led to speculate that vRNA/vaults could be involved in anti-viral defence.

Finally, the hollow structure, the rapid movements, the distinct subcellular localisation (as for example at the nuclear membrane), and the in vitro and clinical correlations with drug resistance in several types of human cancers led to the hypothesis that vaults might represent rather promiscuous transport vehicles [35-37].

### Drug resistance: the best known role of MVP

The MVP has originally been identified as the lung resistance protein [2]. LRP was discovered almost 20 years ago as a protein overexpressed in a multidrug-resistant lung cancer cell line diverting cytostatic drugs from intracellular targets and conferring multidrug resistance (MDR) [19]. Several proteins have been linked to MDR, such as the ABC-drug transporter p-Glycoprotein (P-gp, also designated as ABCB1), members of the multidrug resistant protein family (ABCC1, ABCC2, ABCC3), the human breast cancer resistance protein (BCRP) and the lung-resistance protein (LRP) [36,38,39]. The LRP was initially discovered due to its high expression level in a multidrug-resistant but ABCB1-negative lung cancer cell line [19] and has been identified in 1995 to be the human major vault protein [2]. Thereby, a first link between the MDR and vaults was established.

MVP has been detected in 78% of 61 human cancer cell lines, and its expression levels correlate with resistance against a variety of MDR-related and unrelated drugs [40,41]. Furthermore, upregulation of the MVP expression level was determined in multiple human cancer cell lines also on treatment with anticancer agents, including anthracyclins, etoposide and cisplatin, further supporting a putative link between treatment sensitivity and cellular stress adaptation to cytotoxic agent [19,42-47], and overexpression of MVP is also frequently observed at the early steps of resistance selection [45,46,48,49]. Multiple preclinical studies have corroborated this link between MDR and the MVP expression level in cancer cells, despite some contradicting studies and the fact that the MVP knockout mouse does not show enhanced hypersensitivity to cytostatics. However, this might be due to differential cancer to normal cell line treatment sensitivities or the upregulation of other ABC-transporters or unknown mechanisms of MDR on disruption of functional MVP [11,50].

Vaults may mediate multi-drug resistance by the transport of drugs away from their subcellular targets, by the extrusion from the nucleus and/or the sequestration of drugs into exocytotic vesicles. Either through these exocytotic vesicles or pump molecules like ABCB1, ABCC1, or breast cancer resistance protein at the plasma membrane, the intracellular potency of these cytotoxic agents will be reduced [51]. Alternatively, the two vault-associated VPARP poly(adenosine diphosphate-ribose) polymerase or TEP-1 may fulfil a protective function of the genome and thereby contribute to a drug-resistance profile. Concomitant operation of several drug resistance mechanisms may often be necessary to cause the phenotype of MDR, and co-expression of MVP and ABCC1 frequently occurs and associates with increased drug resistance levels in MDR-selected tumour cells [40]. Interestingly, up-regulation of MVP could be linked in a series of MDR cell lines with a low and intermediate level of drug resistance with subsequent up-regulation of ABCB1 leading to high levels of drug resistance. These findings point the MVP expression level as a marker for low level of drug resistance, which is clinically more relevant [19,45,48]. Furthermore, several studies reported that the expression of MVP is a predictive marker in several tumour types for the response to chemotherapy [52,53].

Interestingly, human vRNAs produce several small RNAs (svRNAs) by mechanisms different from those in the canonical microRNA (miRNA) pathway. svRNAb downregulates CYP3A4, a key enzyme in drug metabolism. This finding contributes to understand the role of vaults particles in drug resistance, proposing a novel function to vRNAs that may help explain this association [54].

### A novel role for MVP: implications in radiotherapy

In recent years, several findings indicate a link between MVP and vaults not only to drug-resistance based on its putative drug-exporter function, but also to DNA-damage and DNA-damage-repair. MVP transcription and protein levels are increased in response to various DNA-damaging agents, including ionizing radiation (IR) [55]. Interestingly, VPARP and, to lesser extent, TEP-1-deficient mice, have an increased incidence of carcinogen-induced colon tumours [56]. These findings indicate that vaults may play a role in facilitating DNA repair processes.

The high cytotoxicity of IR is mainly based on its ability to induce DNA double strand breaks (DSB) via generation of reactive oxygen species. DSB can be repaired by the two major DSB repair machineries: homologous recombination (HR) and non-homologous end joining (NHEJ). The fact that error-free repair by HR depends on the presence of an intact sister chromatid restricts this pathway to occur during S and G2 phases of the cell cycle. This is, in contrast to error-prone repair by NHEJ which can function throughout the cell cycle and is therefore thought to be the predominant DSB repair pathway in mammalian cells. On the other hand, HR represents the more interesting target to sensitize specifically tumor cells to DNA damaging agents leading to an enhanced therapeutic window.

MVP and vaults have recently been linked to both major DSB-machineries. Ku70 and Ku80 are key proteins in NHEJ, and also play a strong regulatory role in apoptosis through Bax/Bcl-2 interactions [57,58]. The DNA end-joining protein Ku70 inhibits apoptosis by sequestering Bax away from the mitochondria. However, newly synthesized Bax undergoes ubiquitination, which negatively regulates its proapoptotic function by labeling it for proteasomal degradation. Interestingly, ubiquitinated Bax associates with Ku70, which mediates Bax deubiquitination, simultaneously generating the active form of Bax but sequestered away from the mitochondria [57]. Low expression level of Ku70 might therefore also result in low expression levels of Bax. On the level of NHEJ, an inverse correlation between high MVP and low expression levels of Ku70/80 and pro-apoptotic bax was identified in a cohort of 160 patients with localized cervix carcinoma. Interestingly, low Ku70/80 expression was also associated with upregulated Bcl-2, altered p53, and increased proliferation in this set of patients [59]. Despite the lack of mechanistic insights these data suggest that overexpression of MVP and vaults might be involved in a carcinogenic process with resultant genomic instability due to suppressed NHEJ. Though enhanced proliferative activity, subsequent tumour progression and enhanced treatment resistance requires at the same time an upregulated apoptotic threshold, which might be activated through the suppression of Bax, upregulation of Bcl-2, mutated p53-status and upregulation of the IGF-1R and downstream signalling cascades. Eventually, only molecular dissection of the key signal transduction cascades leading to reduced NHEJ-activity in MVP-overexpressing and MVP-depleted cells will elucidate the association between MVP/vaults, NHEJ and a deregulated apoptotic threshold. Moreover, MVP increases with age both in vitro and in vivo, and that age-related upregulation of MVP facilitates apoptosis resistance of senescent human diploid fibroblasts based on the interaction with c-Jun-mediated downregulation of BCL-2 [60]. Thus, MVP is suggested to be playing an important role in the resistance of senescent fibroblasts to apoptosis by modulation of BCL-2 expression by JNK pathway, regulating cellular signaling and survival and being a potential therapeutic target for modulation of resistance to apoptosis implicated in aging modulation and cancer treatment [61].

A novel link between the intracellular level of MVP and homologous recombination, the second DNA-DSB-repair machinery, has recently been identified as part of the cellular stress response to ionizing radiation (Presented in ESTRO 29; S 51, 129 oral). MVP-depleted tumour cells are more radiosensitive than their wildtype cognate cells, probably due to direct interference with homologous recombination. In comparison to control cells, Rad51-foci are strongly reduced in irradiated but MVP-depleted cells and thus vaults may coordinate correct HR-complex formation at the site of the DSB. Vaults might also guarantee sufficient protein level of Rad51 required for homologous recombination via coordinated Rad51 expression. It has been reported that MVP mediates nuclear import of PTEN and regulates its nuclear function [24,62]. Nuclear PTEN expression is high in untransformed cells but decreases with tumour progression. While cytoplasmic PTEN inhibits the phosphoinositol-3-kinase (PI3K)/AKT pathway, nuclear PTEN inhibits phosphorylation of MAPK and consequently reduces the expression of cyclin D leading to a G0/G1 cell cycle arrest. Though, PTEN also acts as cofactor for the transcription factor E2F1, driving expression of RAD51. Cells deficient in PTEN have reduced capacity of homologous recombination-dependent DNA repair, most probably due to reduced levels of RAD51. Thereby, PTEN is also important for the maintenance of chromosomal stability reducing the incidence of spontaneous double strand breaks. Interestingly, loss of PTEN in tumour cells and concomitantly reduced homologous recombination can thereby be exploited by the inhibiton of PARP, creating a situation of synthetic lethality [63,64]. Since MVP and vaults are involved in nuclear import of PTEN and homologous recombination, it will be of interest to determine whether the MVP expression level also affects genomic instability and sensitivity to PARP-inhibition.

Despite the fact that we have so far only limited insights into the mechanisms how MVP and vault interact with the two double strand-break repair machineries, these results indicate that MVP and vaults may co-regulate correct DNA repair of spontaneous and treatment-induced double strand breaks. Due to the barrel-like structure vaults may act as carriers not only for toxic agents but also for the coordinated intracellular transport of DNA-repair related proteins and thereby act as scaffold protein or shuttling vector. Otherwise, the vaults associated proteins VPARP or TEP1 might play a so far not-identified role for correct DNA repair, which could only be achieved as part of an intact vaults. As indicated above, in vitro studies revealed that gene expression levels of MDR-related proteins increased after fractionated irradiation. It is well known that radiation treatment can induce resistance to various cytotoxic drugs [65], although the molecular basis of this interaction are complex. Irradiated cell lines showed a significant resistance to cisplatin, doxorubicin and bendamustine, suggesting a novel mechanism in the appearance of MDR, which might involve a radiation-induced increment of LRP/MVP [66].

### MVP and tumour malignancies: vaults as a predictive/prognostic marker

Numerous studies were performed to clarify the expression status of MVP in human malignancies as a predictive and prognostic marker for the chemotherapy response and patient prognosis (Table 1). These studies mainly focused on haematological malignancies. The results should be viewed with caution, since they will be influenced by factors such as 1) MVP detection assays (especially referred to gene expression), 2) number of patients included, or 3) type of statistical analysis performed (univariate vs. multivariate analysis). The quality of MVP as a prognostic or predictive marker in this type of malignancy is still questionable, and standardized detection methods are needed. Nevertheless, a direct association between MVP expression and therapy resistance and prognosis in patients suffering from acute myeloid leukaemia was identified [67-70]. A positive correlation between MVP expression and worse patient prognosis, therapy response, and survival rate has been reported in acute lymphoblastic leukaemia patients [71-73], and similar results have been described in adult T-cell leukaemia [74,75] and multiple myeloma [76-78].

**Table 1:** Clinical studies investigating the association between MVP and therapy response and survival

Author	Patients (n)	Tumour	Response	DFS	OS
Izquierdo [13]	57	Ovarian cancer	Yes	Yes	Yes
Schadendorf [90]	71	Melanoma	Yes		
Ramani [95]	21	Neuroblastoma			No
Dingemans [96]	36	NSCLC	No		No
Linn [97]	70	Breast cancer	No		
Uozaki [98]	60	Osteosarcoma	Yes		Yes
Arts [81]	115	Ovarian cancer	No	No	No
Pohl [99]	99	Breast cancer	No	No	No
Volm [100]	87	NSCLC	Yes		No
Goff [101]	29	Ovarian cancer	No		
Pohl [102]	68	Colorrecto	No		
Diestra [89]	83	Bladder cancer	Yes		
Harada [103]	57	NSCLC	Yes		
Silva [93]	78	HNSCC	Yes		Yes

DFS, disease-free survival; OS, overall survival; NSCLC, non-small cell lung cancer; HNSCC, head-and-neck squamous cell carcinoma.

On the other hand, relatively few studies have addressed the role of MVP in solid tumours, with contradictory findings. Thus, the quality of MVP as prognostic marker in ovarian carcinoma is unclear so far, with positive [79,80] and negative [81,82] associations reported. Result regarding to breast cancer [83,84], non-small cell lung cancer [85,86], or different types of sarcomas are inconclusive [87,88]. On the other hand, MVP has been established as a reliable factor for response to chemotherapy in bladder cancer patients [89], melanoma [90], and determining the aggressive phenotype of testicular germ-cell tumours [91] and glioblastoma [92].

With regard to a putative role of MVP in radiation resistance, it has been reported that MVP expression is strongly associated with local-disease free survival and cancer-specific survival in a series of patients suffering from squamous cell carcinoma of the oropharynx who received primary radiotherapy with curative intent (in univariate and multivariate analyses) [93]. Elevated MVP expression seems to be associated with a RT-resistant subset of patients, proposing MVP as a novel useful prognostic marker associated with RT [93]. The underlying mechanisms behind this association are not well defined today but might be linked to the above cited role of MVP in DNA repair and apoptotic threshold.

The complexity but also interest in MVP as a predictive and/or prognostic marker is also illustrated in cervical cancer patients receiving combined radiochemotherapy [52]. Increased MVP and IGF-1R expression levels were related to a small cohort of cervical cancer patients with reduced long-term local control, in patients who otherwise achieved clinical complete response to radiochemotherapy. High MVP expression was strongly related to high IGF1-R expression, which is associated with chemo- and radioresistance in localized cervical carcinoma patients [94]. This association suggests that both proteins need to be expressed to confer chemoradioresistance in cervical cancer. This was further supported by a subgroup of patients who showed low levels of MVP and IGF1R and who presented excellent survival rates once [52]. All of the responding patients with negative or fairly positive (low MVP) tumours were free of local, distant or death related disease. These results were similar to the ones previously obtained for IGF-1R as a prognostic marker [94]. Combination analysis was performed grouping MVP and IGF-1R expression in tumours, showing that all responding patients with low MVP/IGF-1R tumours were free of local disease, relapse or death related disease.

As illustrated by these studies, we are still far away to have identified a mechanistic link between MVP expression level and treatment response, and it is still a matter of debate in how far vaults are involved in chemo- and radioresistance and thus might serve as predictive marker of therapy response. MVP is associated with MDR, and several studies have recognized MVP as a negative prognostic factor for response to chemo- and radiotherapy and/or disease-free survival or overall survival. Molecular, genetic, and clinical data obtained so far warrant further studies into the role of MVP, vaults-related resistance mechanisms.

## Conclusions

Vaults are ubiquitous ribonucleoprotein complexes involved in a diversity of cellular processes, including multidrug resistance, transport mechanisms, signal transmission and immune response. Nonetheless, new roles have been assigned to MVP in the field of radiotherapy, where vaults have been proposed as a useful prognostic marker associated with radiotherapy resistance. The molecular mechanisms behind this relevant role are unknown, although a possible association with relevant key players in non-homologous endjoining repair and homologous recombination could exist. Additional translational and clinical studies are required to test this hypothesis.

## List of abbreviations

HIF-1: hypoxia-inducible factor-1; HR: homologous recombination; MDR: multidrug resistance; MRP: multidrug resistance protein; MVP: major vaults protein; NHEJ: non-homologous end joining; RT: radiotherapy; TEP-1: telomerase-associated protein-1; VPARP: vault poly(adenosine diphosphate-ribose) polymerase.

## Competing interests

The authors declare that they have no competing interests.

## Authors' contributions

PCL has been involved in revising the manuscript critically for important intellectual content and has given final approval of the version to be published, MP has been involved in drafting the manuscript and has made substantial contributions to conception and design, MZ has made substantial contributions to conception and design, LAHH has been involved in drafting the manuscript and has made substantial contributions to conception and design. All authors read and approved the final manuscript.

## References

[B1] Kedersha NL Heuser JE Chugani DC Rome LH Vaults. III. Vault ribonucleoprotein particles open into flower-like structures with octagonal symmetry J Cell Biol 1991 112 225 235 10.1083/jcb.112.2.225 1988458 PMC2288824

[B2] Scheffer GL Wijngaard PL Flens MJ Izquierdo MA Slovak ML Pinedo HM Meijer CJ Clevers HC Scheper RJ The drug resistance-related protein LRP is the human major vault protein Nat Med 1995 1 578 582 10.1038/nm0695-578 7585126

[B3] Kickhoefer VA Siva AC Kedersha NL Inman EM Ruland C Streuli M Rome LH The 193-kD vault protein, VPARP, is a novel poly(ADP-ribose) polymerase J Cell Biol 1999 146 917 928 10.1083/jcb.146.5.917 10477748 PMC2169495

[B4] Kickhoefer VA Stephen AG Harrington L Robinson MO Rome LH Vaults and telomerase share a common subunit, TEP1 J Biol Chem 1999 274 32712 32717 10.1074/jbc.274.46.32712 10551828

[B5] Kedersha NL Rome LH Isolation and characterization of a novel ribonucleoprotein particle: large structures contain a single species of small RNA J Cell Biol 1986 103 699 709 10.1083/jcb.103.3.699 2943744 PMC2114306

[B6] Tanaka H Kato K Yamashita E Sumizawa T Zhou Y Yao M Iwasaki K Yoshimura M Tsukihara T The structure of rat liver vault at 3.5 angstrom resolution Science 2009 323 384 388 10.1126/science.1164975 19150846

[B7] Xia Y Ramgopal Y Li H Shang L Srinivas P Kickhoefer VA Rome LH Preiser PR Boey F Zhang H Venkatraman SS Immobilization of recombinant vault nanoparticles on solid substrates ACS Nano 2010 4 1417 1424 10.1021/nn901167s 20146454

[B8] Yang J Kickhoefer VA Ng BC Gopal A Bentolila LA John S Tolbert SH Rome LH Vaults Are Dynamically Unconstrained Cytoplasmic Nanoparticles Capable of Half Vault Exchange ACS Nano 2010 4 7229 7240 10.1021/nn102051r 21121616 PMC3020078

[B9] Kong LB Siva AC Kickhoefer VA Rome LH Stewart PL RNA location and modeling of a WD40 repeat domain within the vault RNA 2000 6 890 900 10.1017/S1355838200000157 10864046 PMC1369965

[B10] Kedersha NL Miquel MC Bittner D Rome LH Vaults. II. Ribonucleoprotein structures are highly conserved among higher and lower eukaryotes J Cell Biol 1990 110 895 901 10.1083/jcb.110.4.895 1691193 PMC2116106

[B11] Mossink MH van Zon A Franzel-Luiten E Schoester M Kickhoefer VA Scheffer GL Scheper RJ Sonneveld P Wiemer EA Disruption of the murine major vault protein (MVP/LRP) gene does not induce hypersensitivity to cytostatics Cancer Res 2002 62 7298 7304 12499273

[B12] Slovak ML Ho JP Cole SP Deeley RG Greenberger L de Vries EG Broxterman HJ Scheffer GL Scheper RJ The LRP gene encoding a major vault protein associated with drug resistance maps proximal to MRP on chromosome 16: evidence that chromosome breakage plays a key role in MRP or LRP gene amplification Cancer Res 1995 55 4214 4219 7671223

[B13] Izquierdo MA van der Zee AG Vermorken JB van der Valk P Belien JA Giaccone G Scheffer GL Flens MJ Pinedo HM Kenemans P Drug resistance-associated marker Lrp for prediction of response to chemotherapy and prognoses in advanced ovarian carcinoma J Natl Cancer Inst 1995 87 1230 1237 10.1093/jnci/87.16.1230 7563169

[B14] Lange C Walther W Schwabe H Stein U Cloning and initial analysis of the human multidrug resistance-related MVP/LRP gene promoter Biochem Biophys Res Commun 2000 278 125 133 10.1006/bbrc.2000.3782 11071864

[B15] Schroder K Hertzog PJ Ravasi T Hume DA Interferon-gamma: an overview of signals, mechanisms and functions J Leukoc Biol 2004 75 163 189 14525967 10.1189/jlb.0603252

[B16] Steiner E Holzmann K Pirker C Elbling L Micksche M Sutterluty H Berger W The major vault protein is responsive to and interferes with interferon-gamma-mediated STAT1 signals J Cell Sci 2006 119 459 469 10.1242/jcs.02773 16418217

[B17] Izquierdo MA Scheffer GL Flens MJ Giaccone G Broxterman HJ Meijer CJ van der Valk P Scheper RJ Broad distribution of the multidrug resistance-related vault lung resistance protein in normal human tissues and tumors Am J Pathol 1996 148 877 887 8774142 PMC1861735

[B18] Kedersha NL Rome LH Vaults: large cytoplasmic RNP's that associate with cytoskeletal elements Mol Biol Rep 1990 14 121 122 10.1007/BF00360441 1694556

[B19] Scheper RJ Broxterman HJ Scheffer GL Kaaijk P Dalton WS van Heijningen TH van Kalken CK Slovak ML de Vries EG van der Valk P Overexpression of a M(r) 110,000 vesicular protein in non-P-glycoprotein-mediated multidrug resistance Cancer Res 1993 53 1475 1479 7680954

[B20] Chugani DC Rome LH Kedersha NL Evidence that vault ribonucleoprotein particles localize to the nuclear pore complex J Cell Sci 1993 106 Pt 1 23 29 8270627 10.1242/jcs.106.1.23

[B21] Hamill DR Suprenant KA Characterization of the sea urchin major vault protein: a possible role for vault ribonucleoprotein particles in nucleocytoplasmic transport Dev Biol 1997 190 117 128 10.1006/dbio.1997.8676 9331335

[B22] Kolli S Zito CI Mossink MH Wiemer EA Bennett AM The major vault protein is a novel substrate for the tyrosine phosphatase SHP-2 and scaffold protein in epidermal growth factor signaling J Biol Chem 2004 279 29374 29385 10.1074/jbc.M313955200 15133037

[B23] Kim E Lee S Mian MF Yun SU Song M Yi KS Ryu SH Suh PG Crosstalk between Src and major vault protein in epidermal growth factor-dependent cell signalling FEBS J 2006 273 793 804 10.1111/j.1742-4658.2006.05112.x 16441665

[B24] Yu Z Fotouhi-Ardakani N Wu L Maoui M Wang S Banville D Shen SH PTEN associates with the vault particles in HeLa cells J Biol Chem 2002 277 40247 40252 10.1074/jbc.M207608200 12177006

[B25] Chung JH Ginn-Pease ME Eng C Phosphatase and tensin homologue deleted on chromosome 10 (PTEN) has nuclear localization signal-like sequences for nuclear import mediated by major vault protein Cancer Res 2005 65 4108 4116 10.1158/0008-5472.CAN-05-0124 15899801

[B26] Yi C Li S Chen X Wiemer EA Wang J Wei N Deng XW Major vault protein, in concert with constitutively photomorphogenic 1, negatively regulates c-Jun-mediated activator protein 1 transcription in mammalian cells Cancer Res 2005 65 5835 5840 10.1158/0008-5472.CAN-05-0423 15994960

[B27] Iwashita K Ikeda R Takeda Y Sumizawa T Furukawa T Yamaguchi T Akiyama S Yamada K Major vault protein forms complexes with hypoxia-inducible factor (HIF)-1alpha and reduces HIF-1alpha level in ACHN human renal adenocarcinoma cells Cancer Sci 2010 101 920 926 10.1111/j.1349-7006.2009.01481.x 20175781 PMC11159190

[B28] Kowalski MP Dubouix-Bourandy A Bajmoczi M Golan DE Zaidi T Coutinho-Sledge YS Gygi MP Gygi SP Wiemer EA Pier GB Host resistance to lung infection mediated by major vault protein in epithelial cells Science 2007 317 130 132 10.1126/science.1142311 17615361 PMC3685177

[B29] Mossink MH de Groot J van Zon A Franzel-Luiten E Schoester M Scheffer GL Sonneveld P Scheper RJ Wiemer EA Unimpaired dendritic cell functions in MVP/LRP knockout mice Immunology 2003 110 58 65 10.1046/j.1365-2567.2003.01708.x 12941141 PMC1783015

[B30] Schroeijers AB Reurs AW Scheffer GL Stam AG de Jong MC Rustemeyer T Wiemer EA de Gruijl TD Scheper RJ Up-regulation of drug resistance-related vaults during dendritic cell development J Immunol 2002 168 1572 1578 11823484 10.4049/jimmunol.168.4.1572

[B31] Berger W Steiner E Grusch M Elbling L Micksche M Vaults and the major vault protein: novel roles in signal pathway regulation and immunity Cell Mol Life Sci 2009 66 43 61 10.1007/s00018-008-8364-z 18759128 PMC11131553

[B32] Motsch N Pfuhl T Mrazek J Barth S Grasser FA Epstein-Barr virus-encoded latent membrane protein 1 (LMP1) induces the expression of the cellular microRNA miR-146a RNA Biol 2007 4 131 137 10.4161/rna.4.3.5206 18347435

[B33] Mrazek J Kreutmayer SB Grasser FA Polacek N Huttenhofer A Subtractive hybridization identifies novel differentially expressed ncRNA species in EBV-infected human B cells Nucleic Acids Res 2007 35 e73 10.1093/nar/gkm244 17478510 PMC1904266

[B34] Taganov KD Boldin MP Chang KJ Baltimore D NF-kappaB-dependent induction of microRNA miR-146, an inhibitor targeted to signaling proteins of innate immune responses Proc Natl Acad Sci USA 2006 103 12481 12486 10.1073/pnas.0605298103 16885212 PMC1567904

[B35] Kickhoefer VA Vasu SK Rome LH Vaults are the answer, what is the question? Trends Cell Biol 1996 6 174 178 10.1016/0962-8924(96)10014-3 15157468

[B36] Steiner E Holzmann K Elbling L Micksche M Berger W Cellular functions of vaults and their involvement in multidrug resistance Curr Drug Targets 2006 7 923 934 10.2174/138945006778019345 16918321

[B37] Suprenant KA Vault ribonucleoprotein particles: sarcophagi, gondolas, or safety deposit boxes? Biochemistry 2002 41 14447 14454 10.1021/bi026747e 12463742

[B38] Hall MD Handley MD Gottesman MM Is resistance useless? Multidrug resistance and collateral sensitivity Trends Pharmacol Sci 2009 30 546 556 10.1016/j.tips.2009.07.003 19762091 PMC2774243

[B39] Scheffer GL Schroeijers AB Izquierdo MA Wiemer EA Scheper RJ Lung resistance-related protein/major vault protein and vaults in multidrug-resistant cancer Curr Opin Oncol 2000 12 550 556 10.1097/00001622-200011000-00007 11085454

[B40] Izquierdo MA Shoemaker RH Flens MJ Scheffer GL Wu L Prather TR Scheper RJ Overlapping phenotypes of multidrug resistance among panels of human cancer-cell lines Int J Cancer 1996 65 230 237 10.1002/(SICI)1097-0215(19960117)65:2<230::AID-IJC17>3.0.CO;2-H 8567122

[B41] Laurencot CM Scheffer GL Scheper RJ Shoemaker RH Increased LRP mRNA expression is associated with the MDR phenotype in intrinsically resistant human cancer cell lines Int J Cancer 1997 72 1021 1026 10.1002/(SICI)1097-0215(19970917)72:6<1021::AID-IJC17>3.0.CO;2-7 9378536

[B42] Berger W Elbling L Micksche M Expression of the major vault protein LRP in human non-small-cell lung cancer cells: activation by short-term exposure to antineoplastic drugs Int J Cancer 2000 88 293 300 10.1002/1097-0215(20001015)88:2<293::AID-IJC23>3.0.CO;2-S 11004683

[B43] Berger W Spiegl-Kreinecker S Buchroithner J Elbling L Pirker C Fischer J Micksche M Overexpression of the human major vault protein in astrocytic brain tumor cells Int J Cancer 2001 94 377 382 10.1002/ijc.1486 11745417

[B44] Kickhoefer VA Rajavel KS Scheffer GL Dalton WS Scheper RJ Rome LH Vaults are up-regulated in multidrug-resistant cancer cell lines J Biol Chem 1998 273 8971 8974 10.1074/jbc.273.15.8971 9535882

[B45] Moran E Cleary I Larkin AM Amhlaoibh RN Masterson A Scheper RJ Izquierdo MA Center M O'Sullivan F Clynes M Co-expression of MDR-associated markers, including P-170, MRP and LRP and cytoskeletal proteins, in three resistant variants of the human ovarian carcinoma cell line, OAW42 Eur J Cancer 1997 33 652 660 10.1016/S0959-8049(96)00501-1 9274450

[B46] Verovski VN Van den Berge DL Delvaeye MM Scheper RJ De Neve WJ Storme GA Low-level doxorubicin resistance in P-glycoprotein-negative human pancreatic tumour PSN1/ADR cells implicates a brefeldin A-sensitive mechanism of drug extrusion Br J Cancer 1996 73 596 602 10.1038/bjc.1996.103 8605092 PMC2074337

[B47] Kitazono M Sumizawa T Takebayashi Y Chen ZS Furukawa T Nagayama S Tani A Takao S Aikou T Akiyama S Multidrug resistance and the lung resistance-related protein in human colon carcinoma SW-620 cells J Natl Cancer Inst 1999 91 1647 1653 10.1093/jnci/91.19.1647 10511592

[B48] Versantvoort CH Withoff S Broxterman HJ Kuiper CM Scheper RJ Mulder NH de Vries EG Resistance-associated factors in human small-cell lung-carcinoma GLC4 sub-lines with increasing adriamycin resistance Int J Cancer 1995 61 375 380 10.1002/ijc.2910610317 7729950

[B49] Wyler B Shao Y Schneider E Cianfriglia M Scheper RJ Frey BM Gieseler F Schmid L Twentyman PR Lehnert M Intermittent exposure to doxorubicin in vitro selects for multifactorial non-P-glycoprotein-associated multidrug resistance in RPMI 8226 human myeloma cells Br J Haematol 1997 97 65 75 10.1046/j.1365-2141.1997.52649.x 9136943

[B50] Ikuta K Takemura K Sasaki K Kihara M Nishimura M Ueda N Naito S Lee E Shimizu E Yamauchi A Expression of multidrug resistance proteins and accumulation of cisplatin in human non-small cell lung cancer cells Biol Pharm Bull 2005 28 707 712 10.1248/bpb.28.707 15802814

[B51] Mossink MH van Zon A Scheper RJ Sonneveld P Wiemer EA Vaults: a ribonucleoprotein particle involved in drug resistance? Oncogene 2003 22 7458 7467 10.1038/sj.onc.1206947 14576851

[B52] Lloret M Lara PC Bordon E Rey A Falcon O Apolinario RM Clavo B Ruiz A MVP expression is related to IGF1-R in cervical carcinoma patients treated by radiochemotherapy Gynecol Oncol 2008 110 304 307 10.1016/j.ygyno.2008.04.034 18599112

[B53] van den Heuvel-Eibrink MM Sonneveld P Pieters R The prognostic significance of membrane transport-associated multidrug resistance (MDR) proteins in leukemia Int J Clin Pharmacol Ther 2000 38 94 110 10739113 10.5414/cpp38094

[B54] Persson H Kvist A Vallon-Christersson J Medstrand P Borg A Rovira C The non-coding RNA of the multidrug resistance-linked vault particle encodes multiple regulatory small RNAs Nat Cell Biol 2009 11 1268 1271 10.1038/ncb1972 19749744

[B55] Shimamoto Y Sumizawa T Haraguchi M Gotanda T Jueng HC Furukawa T Sakata R Akiyama S Direct activation of the human major vault protein gene by DNA-damaging agents Oncol Rep 2006 15 645 652 16465425

[B56] Raval-Fernandes S Kickhoefer VA Kitchen C Rome LH Increased susceptibility of vault poly(ADP-ribose) polymerase-deficient mice to carcinogen-induced tumorigenesis Cancer Res 2005 65 8846 8852 10.1158/0008-5472.CAN-05-0770 16204055

[B57] Amsel AD Rathaus M Kronman N Cohen HY Regulation of the proapoptotic factor Bax by Ku70-dependent deubiquitylation Proc Natl Acad Sci USA 2008 105 5117 5122 10.1073/pnas.0706700105 18362350 PMC2278173

[B58] Wang Q Gao F May WS Zhang Y Flagg T Deng X Bcl2 negatively regulates DNA double-strand-break repair through a nonhomologous end-joining pathway Mol Cell 2008 29 488 498 10.1016/j.molcel.2007.12.029 18313386 PMC2806186

[B59] Lloret M Lara PC Bordon E Fontes F Rey A Pinar B Falcon O Major vault protein may affect nonhomologous end-joining repair and apoptosis through Ku70/80 and bax downregulation in cervical carcinoma tumors Int J Radiat Oncol Biol Phys 2009 73 976 979 10.1016/j.ijrobp.2008.11.013 19251084

[B60] Ryu SJ An HJ Oh YS Choi HR Ha MK Park SC On the role of major vault protein in the resistance of senescent human diploid fibroblasts to apoptosis Cell Death Differ 2008 15 1673 1680 10.1038/cdd.2008.96 18600231

[B61] Ryu SJ Park SC Targeting major vault protein in senescence-associated apoptosis resistance Expert Opin Ther Targets 2009 13 479 484 10.1517/14728220902832705 19335069

[B62] Minaguchi T Waite KA Eng C Nuclear localization of PTEN is regulated by Ca(2+) through a tyrosil phosphorylation-independent conformational modification in major vault protein Cancer Res 2006 66 11677 11682 10.1158/0008-5472.CAN-06-2240 17178862

[B63] Dedes KJ Wetterskog D Mendes-Pereira AM Natrajan R Lambros MB Geyer FC Vatcheva R Savage K Mackay A Lord CJ PTEN deficiency in endometrioid endometrial adenocarcinomas predicts sensitivity to PARP inhibitors Sci Transl Med 2010 2 53ra75 10.1126/scitranslmed.3001538 20944090

[B64] Mendes-Pereira AM Martin SA Brough R McCarthy A Taylor JR Kim JS Waldman T Lord CJ Ashworth A Synthetic lethal targeting of PTEN mutant cells with PARP inhibitors EMBO Mol Med 2009 1 315 322 10.1002/emmm.200900041 20049735 PMC3378149

[B65] Mitchell JB Russo A Cook JA Glatstein E Tumor cell drug and radiation resistance: does an interrelationship exist? Cancer Treat Res 1989 48 189 203 10.1007/978-1-4613-1601-5_12 2577138

[B66] Bottke D Koychev D Busse A Heufelder K Wiegel T Thiel E Hinkelbein W Keilholz U Fractionated irradiation can induce functionally relevant multidrug resistance gene and protein expression in human tumor cell lines Radiat Res 2008 170 41 48 10.1667/RR0986.1 18582150

[B67] Filipits M Pohl G Stranzl T Suchomel RW Scheper RJ Jager U Geissler K Lechner K Pirker R Expression of the lung resistance protein predicts poor outcome in de novo acute myeloid leukemia Blood 1998 91 1508 1513 9473213

[B68] Filipits M Stranzl T Pohl G Heinzl H Jager U Geissler K Fonatsch C Haas OA Lechner K Pirker R Drug resistance factors in acute myeloid leukemia: a comparative analysis Leukemia 2000 14 68 76 10.1038/sj.leu.2401634 10637479

[B69] Pirker R Pohl G Stranzl T Suchomel RW Scheper RJ Jager U Geissler K Lechner K Filipits M The lung resistance protein (LRP) predicts poor outcome in acute myeloid leukemia Adv Exp Med Biol 1999 457 133 139 10.1007/978-1-4615-4811-9_15 10500788

[B70] Xu D Arestrom I Virtala R Pisa P Peterson C Gruber A High levels of lung resistance related protein mRNA in leukaemic cells from patients with acute myelogenous leukaemia are associated with inferior response to chemotherapy and prior treatment with mitoxantrone Br J Haematol 1999 106 627 633 10.1046/j.1365-2141.1999.01611.x 10468850

[B71] Oh EJ Kahng J Kim Y Kim M Lim J Kang CS Min WS Cho B Lee A Lee KY Expression of functional markers in acute lymphoblastic leukemia Leuk Res 2003 27 903 908 10.1016/S0145-2126(03)00026-2 12860010

[B72] Valera ET Scrideli CA Queiroz RG Mori BM Tone LG Multiple drug resistance protein (MDR-1), multidrug resistance-related protein (MRP) and lung resistance protein (LRP) gene expression in childhood acute lymphoblastic leukemia Sao Paulo Med J 2004 122 166 171 10.1590/S1516-31802004000400007 15543372 PMC11126162

[B73] Volm M Stammler G Zintl F Koomagi R Sauerbrey A Expression of lung resistance-related protein (LRP) in initial and relapsed childhood acute lymphoblastic leukemia Anticancer Drugs 1997 8 662 665 10.1097/00001813-199708000-00003 9311441

[B74] Ohno N Tani A Uozumi K Hanada S Furukawa T Akiba S Sumizawa T Utsunomiya A Arima T Akiyama S Expression of functional lung resistance--related protein predicts poor outcome in adult T-cell leukemia Blood 2001 98 1160 1165 10.1182/blood.V98.4.1160 11493465

[B75] Sakaki Y Terashi K Yamaguchi A Kawamata N Tokito Y Mori H Umehara M Yoshiyama T Ohtsubo H Arimura K Human T-cell lymphotropic virus type I Tax activates lung resistance-related protein expression in leukemic clones established from an adult T-cell leukemia patient Exp Hematol 2002 30 340 345 10.1016/S0301-472X(02)00775-0 11937269

[B76] Filipits M Drach J Pohl G Schuster J Stranzl T Ackermann J Konigsberg R Kaufmann H Gisslinger H Huber H Expression of the lung resistance protein predicts poor outcome in patients with multiple myeloma Clin Cancer Res 1999 5 2426 2430 10499614

[B77] Rimsza LM Campbell K Dalton WS Salmon S Willcox G Grogan TM The major vault protein (MVP), a new multidrug resistance associated protein, is frequently expressed in multiple myeloma Leuk Lymphoma 1999 34 315 324 10439368 10.3109/10428199909050956

[B78] Schwarzenbach H Expression of MDR1/P-glycoprotein, the multidrug resistance protein MRP, and the lung-resistance protein LRP in multiple myeloma Med Oncol 2002 19 87 104 10.1385/MO:19:2:87 12180485

[B79] Brinkhuis M Izquierdo MA Baak JP van Diest PJ Kenemans P Scheffer GL Scheper RJ Expression of multidrug resistance-associated markers, their relation to quantitative pathologic tumour characteristics and prognosis in advanced ovarian cancer Anal Cell Pathol 2002 24 17 23 12122280 10.1155/2002/958436 PMC4618973

[B80] Wang W Ke S Chen G Gao Q Wu S Wang S Zhou J Yang X Lu Y Ma D Effect of lung resistance-related protein on the resistance to cisplatin in human ovarian cancer cell lines Oncol Rep 2004 12 1365 1370 15547764

[B81] Arts HJ Katsaros D de Vries EG Massobrio M Genta F Danese S Arisio R Scheper RJ Kool M Scheffer GL Drug resistance-associated markers P-glycoprotein, multidrug resistance-associated protein 1, multidrug resistance-associated protein 2, and lung resistance protein as prognostic factors in ovarian carcinoma Clin Cancer Res 1999 5 2798 2805 10537344

[B82] Mayr D Pannekamp U Baretton GB Gropp M Meier W Flens MJ Scheper R Diebold J Immunohistochemical analysis of drug resistance-associated proteins in ovarian carcinomas Pathol Res Pract 2000 196 469 475 10.1016/S0344-0338(00)80048-5 10926324

[B83] Burger H Foekens JA Look MP Meijer-van Gelder ME Klijn JG Wiemer EA Stoter G Nooter K RNA expression of breast cancer resistance protein, lung resistance-related protein, multidrug resistance-associated proteins 1 and 2, and multidrug resistance gene 1 in breast cancer: correlation with chemotherapeutic response Clin Cancer Res 2003 9 827 836 12576456

[B84] Schneider J Lucas R Sanchez J Ruibal A Tejerina A Martin M Modulation of molecular marker expression by induction chemotherapy in locally advanced breast cancer: correlation with the response to therapy and the expression of MDR1 and LRP Anticancer Res 2000 20 4373 4377 11205274

[B85] Berger W Setinek U Hollaus P Zidek T Steiner E Elbling L Cantonati H Attems J Gsur A Micksche M Multidrug resistance markers P-glycoprotein, multidrug resistance protein 1, and lung resistance protein in non-small cell lung cancer: prognostic implications J Cancer Res Clin Oncol 2005 131 355 363 10.1007/s00432-004-0653-9 15856298 PMC12161247

[B86] Chiou JF Liang JA Hsu WH Wang JJ Ho ST Kao A Comparing the relationship of Taxol-based chemotherapy response with P-glycoprotein and lung resistance-related protein expression in non-small cell lung cancer Lung 2003 181 267 273 10.1007/s00408-003-1029-7 14705770

[B87] Komdeur R Klunder J van der Graaf WT van den Berg E de Bont ES Hoekstra HJ Molenaar WM Multidrug resistance proteins in rhabdomyosarcomas: comparison between children and adults Cancer 2003 97 1999 2005 10.1002/cncr.11259 12673730

[B88] Komdeur R Plaat BE van der Graaf WT Hoekstra HJ Hollema H van den Berg E Zwart N Scheper RJ Molenaar WM Expression of multidrug resistance proteins, P-gp, MRP1 and LRP, in soft tissue sarcomas analysed according to their histological type and grade Eur J Cancer 2003 39 909 916 10.1016/S0959-8049(03)00029-7 12706359

[B89] Diestra JE Condom E Del Muro XG Scheffer GL Perez J Zurita AJ Munoz-Segui J Vigues F Scheper RJ Capella G Expression of multidrug resistance proteins P-glycoprotein, multidrug resistance protein 1, breast cancer resistance protein and lung resistance related protein in locally advanced bladder cancer treated with neoadjuvant chemotherapy: biological and clinical implications J Urol 2003 170 1383 1387 10.1097/01.ju.0000074710.96154.c9 14501774

[B90] Schadendorf D Makki A Stahr C van Dyck A Wanner R Scheffer GL Flens MJ Scheper R Henz BM Membrane transport proteins associated with drug resistance expressed in human melanoma Am J Pathol 1995 147 1545 1552 7495278 PMC1869943

[B91] Mandoky L Geczi L Doleschall Z Bodrogi I Csuka O Kasler M Bak M Expression and prognostic value of the lung resistance-related protein (LRP) in germ cell testicular tumors Anticancer Res 2004 24 1097 1104 15154630

[B92] Tews DS Nissen A Kulgen C Gaumann AK Drug resistance-associated factors in primary and secondary glioblastomas and their precursor tumors J Neurooncol 2000 50 227 237 10.1023/A:1006491405010 11263502

[B93] Silva P West CM Slevin N Valentine H Ryder WD Hampson L Bibi R Sloan P Thakker N Homer J Hampson I Tumor expression of major vault protein is an adverse prognostic factor for radiotherapy outcome in oropharyngeal carcinoma Int J Radiat Oncol Biol Phys 2007 69 133 140 10.1016/j.ijrobp.2007.02.025 17459603

[B94] Lloret M Lara PC Bordon E Pinar B Rey A Falcon O Molano F Hernandez MA IGF-1R expression in localized cervical carcinoma patients treated by radiochemotherapy Gynecol Oncol 2007 106 8 11 10.1016/j.ygyno.2007.04.004 17490736

[B95] Ramani P Dewchand H Expression of mdr1/P-glycoprotein and p110 in neuroblastoma J Pathol 1995 175 13 22 10.1002/path.1711750104 7891222

[B96] Dingemans AM van Ark-Otte J van der Valk P Apolinario RM Scheper RJ Postmus PE Giaccone G Expression of the human major vault protein LRP in human lung cancer samples and normal lung tissues Ann Oncol 1996 7 625 630 8879378 10.1093/oxfordjournals.annonc.a010681

[B97] Linn SC Pinedo HM van Ark-Otte J van der Valk P Hoekman K Honkoop AH Vermorken JB Giaccone G Expression of drug resistance proteins in breast cancer, in relation to chemotherapy Int J Cancer 1997 71 787 795 10.1002/(SICI)1097-0215(19970529)71:5<787::AID-IJC16>3.0.CO;2-5 9180147

[B98] Uozaki H Horiuchi H Ishida T Iijima T Imamura T Machinami R Overexpression of resistance-related proteins (metallothioneins, glutathione-S-transferase pi, heat shock protein 27, and lung resistance-related protein) in osteosarcoma. Relationship with poor prognosis Cancer 1997 79 2336 2344 10.1002/(SICI)1097-0142(19970615)79:12<2336::AID-CNCR7>3.0.CO;2-J 9191521

[B99] Pohl G Filipits M Suchomel RW Stranzl T Depisch D Pirker R Expression of the lung resistance protein (LRP) in primary breast cancer Anticancer Res 1999 19 5051 5055 10697509

[B100] Volm M Rittgen W Cellular predictive factors for the drug response of lung cancer Anticancer Res 2000 20 3449 3458 11131647

[B101] Goff BA Paley PJ Greer BE Gown AM Evaluation of chemoresistance markers in women with epithelial ovarian carcinoma Gynecol Oncol 2001 81 18 24 10.1006/gyno.2000.6105 11277644

[B102] Pohl G Suchomel RW Stranzl T Depisch D Stiglbauer W Filipits M Pirker R Expression of the lung resistance protein in primary colorectal carcinomas Anticancer Res 2001 21 201 204 11299735

[B103] Harada T Ogura S Yamazaki K Kinoshita I Itoh T Isobe H Yamashiro K Dosaka-Akita H Nishimura M Predictive value of expression of P53, Bcl-2 and lung resistance-related protein for response to chemotherapy in non-small cell lung cancers Cancer Sci 2003 94 394 399 10.1111/j.1349-7006.2003.tb01453.x 12824911 PMC11160169

